# A memetic dynamic coral reef optimisation algorithm for simultaneous training, design, and optimisation of artificial neural networks

**DOI:** 10.1038/s41598-024-57654-2

**Published:** 2024-03-23

**Authors:** Francisco Bérchez-Moreno, Antonio M. Durán-Rosal, César Hervás Martínez, Pedro A. Gutiérrez, Juan C. Fernández

**Affiliations:** 1 Department of Computer Science and Numerical Analysis, University of Córdoba, Córdoba, Spain; 2 Department of Quantitative Methods, Universidad Loyola Andalucía, Córdoba, Spain; 3 Maimonides Biomedical Research Institute of Córdoba, IMIBIC, University of Córdoba, 14071 Córdoba, Spain

**Keywords:** Artificial neural networks, Neuroevolution, Coral reef optimisation algorithm, Local search, Classification, Robust estimators, Computational science, Computer science, Scientific data

## Abstract

Artificial Neural Networks (ANNs) have been used in a multitude of real-world applications given their predictive capabilities, and algorithms based on gradient descent, such as Backpropagation (BP) and variants, are usually considered for their optimisation. However, these algorithms have been shown to get stuck at local optima, and they require a cautious design of the architecture of the model. This paper proposes a novel memetic training method for simultaneously learning the ANNs structure and weights based on the Coral Reef Optimisation algorithms (CROs), a global-search metaheuristic based on corals’ biology and coral reef formation. Three versions based on the original CRO combined with a Local Search procedure are developed: (1) the basic one, called Memetic CRO; (2) a statistically guided version called Memetic SCRO (M-SCRO) that adjusts the algorithm parameters based on the population fitness; (3) and, finally, an improved Dynamic Statistically-driven version called Memetic Dynamic SCRO (M-DSCRO). M-DSCRO is designed with the idea of improving the M-SCRO version in the evolutionary process, evaluating whether the fitness distribution of the population of ANNs is normal to automatically decide the statistic to be used for assigning the algorithm parameters. Furthermore, all algorithms are adapted to the design of ANNs by means of the most suitable operators. The performance of the different algorithms is evaluated with 40 classification datasets, showing that the proposed M-DSCRO algorithm outperforms the other two versions on most of the datasets. In the final analysis, M-DSCRO is compared against four state-of-the-art methods, demonstrating its superior efficacy in terms of overall accuracy and minority class performance.

## Introduction

Artificial Neural Networks (ANNs) are widely used in many fields of study such as business, industry, medical diagnosis, Unmanned Aerial Vehicle (UAV) detection and so on^1^. Hence, ANNs have been an object of interest among researchers in areas where standard regression models and other related statistical techniques have traditionally been used.

The Multilayer Perceptron (MLP) with Sigmoidal transfer functions (SUs)^2^ is the most popular and traditional ANN for classification and regression purposes. ANN models need to be trained with data and learning algorithms before making generalisations in classification or regression tasks. Learning algorithms are divided into two main groups: local search (LS) and global search algorithms. The Backpropagation algorithms (BPs)^3^ or Extreme Learning Machines (ELM)^4^ belong to the first group, commonly used for weight optimisation, while Evolutionary Algorithms (EAs)^5^ belong to the second group. This second group is usually referred to Neuroevolution^6^ or application of metaheuristics such as EAs to the evolution of ANNs, also known in the literature as Evolutionary Artificial Neural Networks (EANNs)^7–9^, so that both the weights and the ANN architecture are optimised. Using EAs is an efficient tool because finding a suitable ANN architecture is a controversial topic in Machine Learning (ML), requiring a lot of trial and error procedures and a great deal of experience of the researcher^2^.

On the one hand, growing networks or constructive algorithms^10,11^ are one possible option to obtain the appropriate architecture of an ANN, which starts with a small network, usually a single unit. This network is trained until it is incapable of further learning. This procedure is repeated until a good solution is found. Additionally, destructive methods are also known as pruning algorithms^12^, which begin with an extensive network that usually ends in over-fitting. Then, some procedures are applied to remove the connections and nodes that are not needed. However, these two methodologies are based on the traditional BP and usually suffer from slow convergence. Moreover, as it is well known, the main drawback of classical ANNs training methods is that they can fall into local optima, and the learning process can become stagnant.

On the other hand, Neuroevolution global search methods^13–15^ are used to evolve weights, topologies, parameters and learning strategies of ANNs. They need many generations to reach a good solution, and they are too poor to find the best solution in a region where the algorithm converges toward a global optimum. Therefore, combining EAs and LS procedures would conduct a global search inside the solutions space, locating the ANNs near the global optimum so the local procedure could arrive at the best solution quickly and efficiently. This combination is known in the literature as Memetic Algorithms (MAs)^16,17^.

The use of metaheuristics in ML enables efficient exploration of large solution spaces and the discovery of high-quality solutions for complex optimization problems^15,18,19^. The Coral Reef Optimisation algorithm (CRO) is an evolutionary-type meta-heuristic recently proposed^20,21^. It draws inspiration from the processes observed in real coral reefs, including coral reproduction, depredation, and competition for space^22,23^. The CRO combines elements of both an evolutionary algorithm and a Simulated Annealing approach^21^, yielding impressive results in various challenging optimisation problems^24–26^. Unlike some other meta-heuristics, the CRO is specifically designed with a exploitation perspective (rather than exploration), resulting in different algorithm variants based on the specific search procedures employed. This makes the algorithm particularly well-suited for optimising ANNs, although it has not been previously evaluated for this task. However, the main problem with these algorithms is that they need to establish a high number of parameters to guide the evolutionary process, so the design of an algorithm that automatically configures these parameters can be worthwhile. Regarding LS methodologies, gradient-descent techniques are the most widely used for supervised learning in ANNs. Specifically, the Improved Resilient Backpropagation algorithm (*i*Rprop*+*)^27^ is one of the best techniques known for weight optimisation in terms of convergence speed, accuracy and robustness with respect to its parameters. *i*Rprop*+* algorithm applies a backtracking strategy, and it decides whether to take a step back along a weight direction. Using this LS technique together with a CRO algorithm^28^ would provide a memetic strategy for the design of ANNs.

Several Memetic EANNs using CROs have been developed in this work, but before describing them, it is necessary to introduce the Fitness Landscape (FL) concept. It was first defined by^29^ to demonstrate the dynamics of biological evolutionary optimisation, proving its effectiveness for analysing EAs^30^. A simple definition for that concept is that an FL is a graph where the points in the abscissa axis represent the different individual genotypes in a search space, and the points in the ordinate axis represent the fitness of each one of these individuals^31^. FL is often used to define the dynamics of metaheuristics in optimisation tasks and is usually a statistical description problem^32^. An FL is formed by the fitness function values of all the individuals in the search space, and a neighbourhood search operator is used to calculate the local FL statistics indicators. Some metrics have been conceived to analyse and evaluate the different features of problems, for example, information entropy measure and length scale, among others^6,33^. Many developed techniques are used to analyse it, but only some are applied in practice because FL analysis is complex. FL has been used with EAs operators to select strategies and decide approaches in the evolutionary process^34^. Despite the complexity of FL analysis, it has been performed on artificial combinational benchmarks such as Travelling Salesman Problem, Quadratic Assignment Problem^35^, on artificial numerical problems^36^ or, more recently, on improving the convergence of a genetic-backpropagation algorithm for training ANNs^37^. Studying the use of robust estimators for the analysis of FL can be interesting to adapt the parameters through an evolutionary process automatically.

After reviewing the literature, no works use CRO metaheuristic for the training, design and optimisation of ANNs, all simultaneously relieving the researcher from setting the parameters of CROs. For example, in^38^, time series prediction is carried out with ANN models using a CRO algorithm but with a fixed and fully connected architecture of each network, i.e. only the link weights vary during evolution. Furthermore, in that work, it is necessary to establish all the parameters on which a CRO algorithm depends. In^39^, a CRO algorithm is used to extract the most suitable features in a prediction problem, in this case, Global Solar Radiation (GSR), and then an Extreme Learning Machine (ELM) model is used to obtain the prediction, again having to set the appropriate parameters for the CRO extractor algorithm. In^40^, another GSR prediction problem is solved. In contrast, a CRO algorithm evolves the weights of a neural network in order to improve the solutions obtained, and again with the need to set the parameters suitable for CRO algorithm. In^41^, a statistical version of CRO (SCRO) is developed for reducing the number of elements in time series with minimum information loss, with specific applications on time series segmentation. SCRO is used for time series segmentation based on minimising the error of the piecewise linear approximations (PLAs) obtained for each segment.

This study introduces three Memetic variations of CROs aimed at simultaneously training and designing the topology and weights of ANNs, each utilising the *i*Rprop*+* algorithm as the LS procedure:

- *M-CRO* This version has been developed and adapted for training and designing ANNs using the standard CRO algorithm^20^. The basic version presents the problem of the establishment of multiple parameters for selecting the individuals used with the operators of the evolutionary process. These parameters include the ratio of empty positions in the initialisation phase, the percentage of corals in sexual and asexual reproduction, or the number of less healthy corals that must die to allow empty positions for the next generation of evolution (stages of the evolutionary process), among others.
- *M-SCRO* This version improves the setting of parameters, and it is based on the Statistically-driven CRO (SCRO) algorithm^41^. M-SCRO automatically adjusts the parameters of the standard CRO algorithm, eliminating the need for extensive experimental procedures. M-SCRO selects the corals involved in the operators at different stages of the evolutionary process based on each individual’s fitness and the average fitness of the entire reef. This version assumes that the FL of the population follows a normal distribution.
- *M-DSCRO* The third version of the algorithm is called Memetic Dynamic SCRO (M-DSCRO). M-DSCRO studies the fitness of the entire reef throughout the evolutionary process, checking whether the distribution is normal or not. It does not assume that the FL of the population always follows a normal distribution. The corals involved in each algorithm stage will vary depending on FL. The selection of corals will be based on either the mean and standard deviation of the population or the median and interquartile range, depending on whether the population follows a normal distribution or not. Therefore, MD-SCRO also avoids adjusting multiple algorithm parameters.

The contributions of this paper are briefly summarised below:

- Three versions of the CRO metaheuristic for the simultaneous training, design and optimisation of ANNs. We have hybridised the algorithms (M-CRO, M-SCRO and M-DSCRO) using the *i*Rprop*+* algorithm as LS procedure. To the best of our knowledge, this metaheuristic has not been used before for the purpose of designing and optimising ANNs. The LS procedure is added at the three reproductive stages of the reef. Additionally, a method for reinitialising the reef in case of possible stagnation is included. This is concisely described in the following sections of this work.
- Adaptation of evolutionary operators used with ANNs to the reproduction scheme presented by CRO metaheuristics. The implementation of specific operators is crucial for the optimisation of ANNs using EAs^42–44^. In this work, we have identified crossover and mutation operators used in ANNs that are appropriate for integration into the scheme of a CRO algorithm, more specifically for the sexual and asexual reproduction stages.
- The use of robust estimators instead of assuming normality of the fitness distribution during the evolutionary process. Thanks to them, M-DSCRO enhances the performance of M-CRO and M-SCRO algorithms. The algorithm also automatically determines the individuals to be used in each stage of the evolutionary process, eliminating the need for researchers to establish these parameters.
- The M-SCRO and M-DSCRO algorithms improve the accuracy compared to the basic version, eliminating the need for parameter setting. A statistical study with 40 classification datasets has been carried out to compare the three developed algorithms and other state-of-the-art methodologies in pattern classification. The study considers global accuracy metrics as well as accuracy per class. The results indicate that M-DSCRO outperforms other methodologies on most datasets.

The rest of the paper is organised as follows: “Background and evolutionary stages” section includes the main phases or stages of the evolutionary process of the three algorithms, together with the strategies used in the selection of individuals in M-CRO and M-SCRO algorithms. It continues referencing the LS algorithm used to exploit solutions in the evolutionary process, a description of the reef restarting procedure, the use of the operators adapted to ANNs, and it concludes with a brief introduction to the theory of robust estimators. “M-DSCRO algorithm” section describes in detail the FL strategy used in the M-DSCRO algorithm and the reproductive stages. “Experiments” section shows information about the datasets used in the experimentation, the ANN models used, the metrics employed to evaluate the performance of the algorithms and the experimental design. “Results and discussion” section discusses the results, including their statistical analysis. Finally, the conclusions are shown in “Conclusions” section.

## Background and evolutionary stages

This section presents in a general way the stages of the three Memetic CRO algorithms developed. It continues with a reference to the *i*Rprop*+* algorithm used as an LS optimisation method, an explanation about the reef restarting procedure in case of premature convergence and low diversity, a brief description of the problem of using crossover operators in ANNs, and it concludes with a theoretical explanation of robust estimators used in the M-DSCRO algorithm.

Essentially, the evolutionary stages present in all three algorithms are similar. Hence, this section presents both the shared stages and the distinctions in parameter configuration methods. This encompasses the manual parameter definition for each stage of the M-CRO algorithm, as well as the automatic setup utilised in the M-SCRO algorithm. M-DSCRO also employs automated parameter configuration, which will be introduced within the algorithm’s pseudocode in “M-DSCRO algorithm” section.

**Figure 1:**
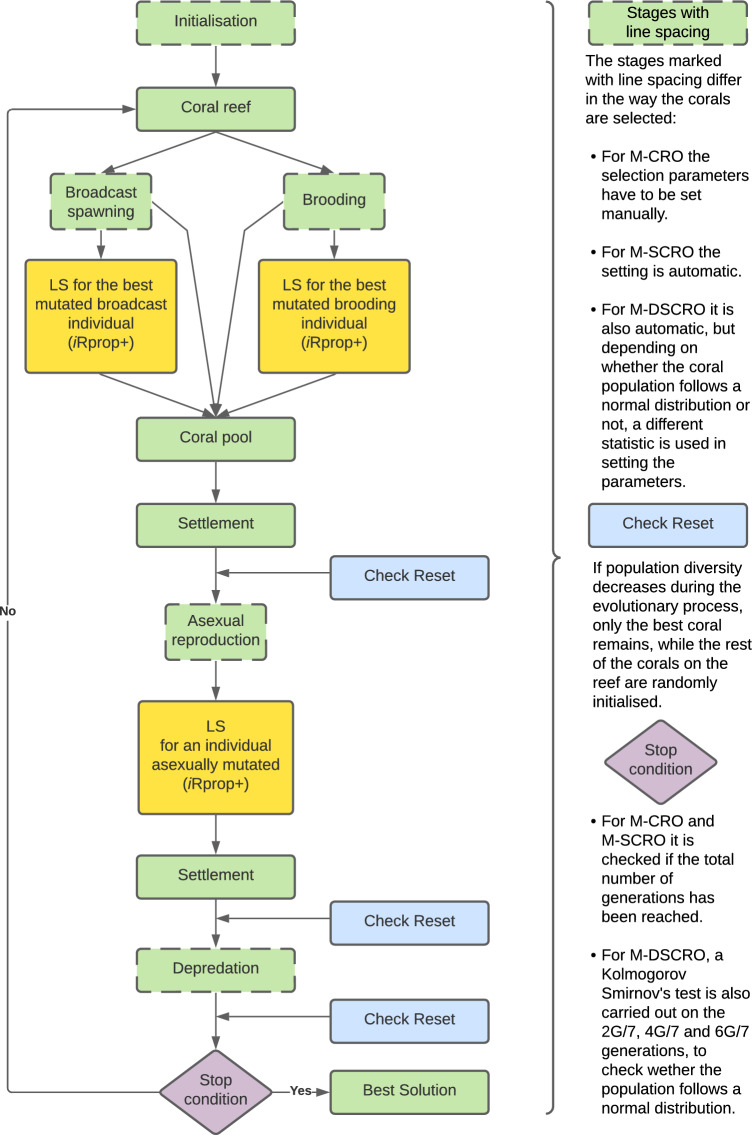
Flowchart of the three Memetic CRO algorithms. The “Background and evolutionary stages” section and the “M-DSCRO algorithm” section describe in detail each of these stages.

Figure 1 illustrates the stages of M-CRO, M-SCRO and M-DSCRO algorithms summarising the procedures discussed below.

The use of CRO methodologies to design and optimise ANNs combines Evolutionary Algorithms and Simulated Annealing. Additionally, the dynamic version (M-DSCRO) eliminates the need for researchers to establish several metaheuristic parameters by analysing the fitness landscape of the population during the evolutionary process.

### Understanding M-CRO and the statistical version, M-SCRO

Standard CRO algorithm^20^ is a type of EA that, by simulating the biological processes occurring in a coral reef, tries to solve search and optimisation problems. In general, each position $c_{i,j}$ of a reef formed by *P* individuals organised in a $P_1 \times P_2$ matrix is a possible solution of the problem to be solved, where $P_1$ and $P_2$ are the number of rows and columns, respectively.

On the other hand, the SCRO algorithm^41^ with self-adaptive parameters was proposed for time series segmentation problems with the idea of removing the high number of parameters needed to be set in the standard CRO. For further understanding of SCRO, let us define two significant variables of this algorithm. Taking into account that the quality of the coral is measured by a fitness function *f* (see Eq. 19), we can define the fitness values of the $N_j$ corals in the *j*-th generation of the algorithm as $\{ f_{1j},f_{2j},\dots ,f_{Nj}\}$. Assuming that the fitness distribution is normal, the variance of the population in the *j*-th generation can be estimated as:

1 $$\begin{aligned} S_{f_j}^2 = \frac{\sum _{i=1}^{N_j} (f_{ij} - \bar{f}_j)^2}{N_j - 1}, \;\; j=1,\dots ,G, \end{aligned}$$

where *G* is the total number of generations, $f_{ij}$ is the fitness of the *i*-th individual in *j*-th generation, and $\bar{f_j}$ is the average fitness value of all the individuals of the generation, expressed as:

2 $$\begin{aligned} \bar{f_j} = \sum _{i=1}^{N_j}f_{ij} / N_j. \end{aligned}$$

Considering the Eqs. (1) and (2), SCRO avoids assigning multiple parameters at different stages of the evolutionary process.

Both M-CRO and M-SCRO are based on the considerations that have just been described and that are clarified in the following subsections.

#### Initialisation in M-CRO and M-SCRO

M-CRO algorithm starts by initialising a random subset of positions from the total number of individuals *P*, leaving the remaining positions empty. The percentage of initial free positions in the coral is determined by a parameter $\rho$ with $0< \rho < 1$, which indicates the ratio of the reef that remains empty initially. The idea is that these positions allow for the settlement and growth of corals in the later stages of the algorithm.

To avoid the parameter $\rho$, the M-SCRO algorithm initialises a complete coral reef with *P* positions, and then, those corals whose fitness $f_{i1} \not \in (\bar{f}_1 - S_{f_1}, 1]$ are deleted. Thus, the parameter $\rho$ is unnecessary. If $(\bar{f}_1 - S_{f_1}) \le 0$ no corals are removed from the reef.

Once the initialisation stage has been performed, the evolutionary block of the algorithms simulates the processes of reproduction and reef formation, using different operators sequentially applied over a number of generations.

#### Sexual reproduction plus Local Search in M-CRO and M-SCRO

Within sexual reproduction, two processes can be distinguished: broadcast spawning (also called external sexual reproduction) and brooding (also called internal sexual reproduction).

For the M-CRO algorithm, in each *i*-th iteration, the broadcast spawning procedure selects a uniform random fraction $F_b$ of corals to be broadcast spawners. To form a new larva, a crossover operator is usually applied. “Crossover operator in artificial neural networks” section explains the problem of using crossover operators for ANNs. On the other hand, the remaining subset of corals on the reef ($1 - F_b$) simulates reproduction in hermaphroditic corals using the brooding operator. Each coral mutates to generate a new larva that becomes part of the candidate solution pool.

M-SCRO algorithm does not need to configure these parameters. Instead, the corals with a fitness function in the interval $f_{ij} \in (\bar{f}_j - S_{f_j}, 1]$ are selected for broadcast spawning. Brooding is applied to the remaining ones, i.e. those whose fitness $f_{ij} \in [0, \bar{f}_j - S_{f_j}]$. Therefore, $F_b$ is not necessary.

At this stage of sexual reproduction, an optimisation procedure is also applied to the best individuals resulting from the Broadcast Spawning and Brooding operators. This optimisation is applied using *i*Rprop*+* as LS algorithm (detailed in “iRprop+ local search algorithm” section). Therefore, the best individual resulting from Broadcast Spawning is optimised with *i*Rprop*+* and added to the Coral Pool, and the same goes for the best individual obtained from Brooding.

#### Coral pool in M-CRO and M-SCRO

The corals obtained from two types of sexual reproduction and asexual reproduction, detailed below, are stored in a coral pool (emptied for each generation), along with the two optimised individuals with the LS algorithm, so that they are the individuals that will be considered for the Settlement stages. All individuals in the pool are evaluated prior to the Settlement stages.

#### Settlement in M-CRO and M-SCRO

Once the sexual and asexual reproduction procedures have been completed, each larva in the candidate pool attempts to settle and grow at a random position (*i*, *j*) on the reef ($P_1 \times P_2$). The larva will be set if the position is empty or if it is healthier than the existing coral at that position, i.e. its fitness value is better. In addition, a maximum number $\nu$ of attempts is established for the larva to search for a feasible position. A robust value for $\nu$ is 2, i.e. a larva has two attempts to settle on the reef^41^.

#### Asexual reproduction plus local search in M-CRO and M-SCRO

For the M-CRO algorithm, the asexual reproduction mimics the reproduction of corals by budding or fragmentation. The mechanism consists of i) ranking corals according to their fitness value, ii) selecting a small fraction $F_a$ of the best corals (we have verified that the performance obtained by choosing a random solution instead of a fraction is similar), iii) duplicating the fraction of best corals to ensure its survival and add to a new candidate pool, and iv) settling the corals from the candidate pool.

In M-SCRO algorithm, in order to eliminate the $F_a$ parameter, a random solution from the set of corals whose fitness verifies $f_{ij} \in (\bar{f}_j + S_{f_j}, 1]$ is selected to be asexually reproduced. If $(\bar{f}_j + S_{f_j}) \ge 1$, asexual reproduction is not carried out.

After asexual reproduction, a mutated individual is randomly selected and duplicated, then the LS is applied. Next, another Settlement process is carried out, so that the optimised individual attempts to establish itself in the coral. We have experimentally verified that the results obtained are similar regardless of the fact that the individual is chosen randomly or the best one is chosen.

#### Depredation in M-CRO and M-SCRO

Finally, the M-CRO algorithm applies a depredation procedure for a percentage $F_d$ of the worst corals in the reef under a given probability $P_d$. It simulates the death of less healthy corals to allow empty positions for the next generation of evolution.

In the case of M-SCRO algorithm, it eliminates the set of individuals whose fitness function verifies $f_{ij} \in [0, \bar{f}_j - 2S_{f_j}]$. In this case, the parameters $F_d$ and $P_d$ do not need to be configured. If $(\bar{f}_j - 2S_{f_j}) \le 0$, depredation is not carried out.

#### Stop condition

The stopping condition of the algorithms is met when a maximum number of generations has been reached.

### *i*Rprop*+* local search algorithm

In this work, the algorithm *i*Rprop*+* is used in the three algorithms implemented as an LS procedure to establish a balance between the exploration and exploitation of the population of ANNs. *i*Rprop*+* is an improvement of the well-known BP algorithm, and its good performance for ANNS weight optimisation has already been proven^27,45^. This algorithm employs a sign-based scheme to update the weights in order to eliminate influences of the derivatives’ magnitude on the weight updates, but it applies a backtracking strategy to decide whether to take a step back along a weight direction by using a heuristic.

In^45^, the reader can see a detailed description of the *i*Rprop*+* adapted to the *softmax* activation function and the cross-entropy error function, used to discern the output class provided by each ANN and as optimisation function respectively. The error and fitness function used in this work can be consulted in “Evaluation metrics” section.

As seen above, the LS is applied to only three individuals in each generation. We have verified that involving more individuals would enormously increase the computational cost, and the results would not be better.

### Reef restarting procedure

By incorporating an LS algorithm during the reproductive stages on the coral reef, it may be the case that the diversity of the population suffers a quick reduction during the search procedure, obtaining a premature convergence to a local optimum. To prevent this possibility, we have introduced in the three algorithms a reef restarting after each stage of reproduction (Sexual, Asexual and Depredation).

The restarting procedure is similar to the Initialisation stage. However, in this case, the best coral is maintained, and the rest of the corals in the reef are randomly initialised. Empty spaces are carried out according to the procedure described in the Initialisation subsections of the three algorithms.

The reef is restarted if one of these two conditions is reached:

- The difference in fitness, $bw_j$, between the best coral $(f_{bj})$ and the worst one $(f_{wj})$ is lower than a threshold, named $t_a$:
  3 $$\begin{aligned} (bw_j=(f_{bj}-f_{wj})) \le t_a \end{aligned}$$
- The $S_{f_j}^2$ value of the population is lower than a threshold, named $t_b$:
  4 $$\begin{aligned} S_{f_j}^2 \le t_b \end{aligned}$$

### Crossover operator in artificial neural networks

A crossover in genetic algorithms or EAs is commonly seen, but certain drawbacks prevent establishing a crossover operator if the EA considers ANNs as individuals^42,46^. The cause is that crossover is impractical in environments where the fitness of an individual in the population is not correlated with the expected ability of its representational components. Such environments are called deceptive^47,48^. Deception is a significant feature in most representations of ANNs, so crossover should be avoided in EANNs^42^.

Therefore, a problem arises when crossing networks with similar structures and weights. That could lead to offspring that contain repeated components in their structure, and therefore, the ability of those components in the parents to be lost. Or it could also happen that the descendant individual was identical to one of the parents since changing the order nodes, we do not alter the individual itself (problem of deception^42^). Thus, the result would be, in either case, an offspring with individuals equal to or worse than their parents, so the crossover operator is useless.

A similar drawback occurs when crossing two networks with the same structure but different weights. Each hidden node plays a specific role in each ANN, and it is the set of hidden nodes that have evolved together that makes a network have a good fitness. Therefore, crossing several random neurons from different networks to create offspring will not generate good individuals.

Finally, crossing two networks with different structures will surely be incompatible, reducing the possibility of producing offspring, so if successful, good individuals are unlikely to be generated.

Therefore, it is a complicated task to generate a crossover operator for EAs with ANNs since these drawbacks must be taken into account and compensated in some way to generate good offspring from the crossover of two individuals.

For this reason, only two types of mutations have been used for the development of the M-CRO, M-SCRO and M-DSCRO algorithms in their sexual reproduction stages: structural and parametric mutations^49,50^. It is detailed below.

#### Operators used in the sexual reproduction stages

Taking into account the problems of using the crossover operator in ANNs discussed above, this subsection describes the mutation operators used in the sexual reproduction stage of the implemented algorithms. The types of mutations applied are the same for the M-CRO, M-SCRO and M-DSCRO algorithms.

For broadcast spawning, structural mutations are applied (it explores the search space). Structural mutations affect the topology of an ANN and allow different regions in the search space to be explored. This type of mutation modifies the number of hidden neurons and the number of links between neurons in the hidden layer, as well as the number of neurons in the input and output layers. Note that mutations implemented for the developed algorithms are: nodes deletion, connections deletion, nodes addition, connections addition and nodes fusion.

**Figure 2:**
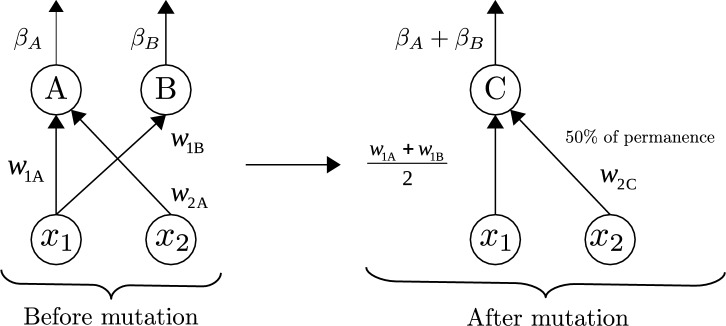
Mutation *node fusion*. A and B are the initial neurons involved in the fusion, and C is the neuron resulting from the mutation.

The mutations *add or delete neurons* consist of randomly adding or removing a minimum and maximum number of neurons. For deleted neurons, their links are also deleted. And for added neurons, the links are randomly established with a value according to an interval. The mutation *node fusion* randomly choose two neurons, A and B, and replace them with another new neuron, C. The connections from neuron C to the nodes shared by neurons A and B will be preserved. Additionally, those that are not common will also be kept with a probability of 0.5 (see example in Fig. 2).

In the mutations *add or delete links*, the number of links to add or delete is applied between the input and the hidden layer and between the hidden and the output layer.

For brooding, parametric mutations are applied (it exploits the search space). The parametric mutation modifies the model coefficients aggregating Gaussian noise, using a self-adaptive annealing process^51,52^. The variance of the Gauss distribution depends on the temperature factor based on the aptitude (see “Evaluation metrics” section) of each individual *i*, which will decrease along the evolutionary way to avoid aggressive mutations at the end of that process:

5 $$\begin{aligned} T(i) = 1 - A(i), \qquad 0 \le T(i) < 1 \end{aligned}$$

being *A*(*i*) the aptitude of the individual.

Specifically, the parametric mutation affects the weights $w_{kl}$ (weight for the *k*-th input of the *l*-th neuron of the hidden layer) and $\beta _{lq}$ (weight for the *l*-th hidden neuron of the *q*-th neuron of the output layer) of the network as follows:

6 $$\begin{aligned} w_{kl}(j+1)&= w_{kl}(j) + \epsilon _{1}(j), \end{aligned}$$

7 $$\begin{aligned} \beta _{lq}(j+1)&= \beta _{lq}(j) + \epsilon _{2}(j), \end{aligned}$$

where $\epsilon _{1}(j)$ and $\epsilon _{2}(j)$ represents one dimensional normally distributed random value from $N(0, \alpha _{1}(j)T(i))$ or $N(0, \alpha _{2}(j)T(i))$ respectively, and where $\alpha _{1}(j)$ and $\alpha _{2}(j)$ are parameters that together with the temperature determines the variance of the distribution, which varies during evolution adapting the learning process, and *j* is the generation number in the evolutionary process.

### Robust estimators

In the above-mentioned M-SCRO algorithm, the distribution of the fitness function is assumed to be normal throughout the evolution, using the mean as the centralisation statistic and the standard deviation as the dispersion statistic. If the fitness distribution is not normal, it is necessary to change these statistics. The use of these other estimators (fully described in “Selection of individuals in M-DSCRO” section) relies on the theory of robust estimators. A robust estimator is fully efficient for an assumed distribution but maintains high efficiency for plausible alternatives^53,54^. The robustness property can be studied through the breakpoint and the influence function of any estimator.

Robust statistics is an area of mathematical statistics that appeared in the 1960s. Its foundations include mainly three works^53,55,56^, which are the basis of later studies on these statistics^57^.

One type of robust estimator is *M*-estimators, a generalisation of the estimators acquired by the maximum likelihood method, whose objective function is a sample average^58^. Thus, given a sample and a function $\psi$, *T* is said to be an *M*-location estimator based on the function $\psi$ if:

8 $$\begin{aligned} \sum _{i=1}^{n}\psi (x_{i} - T) = 0. \end{aligned}$$

According to the previous expression, the sample mean is a function-based *M*-estimator as well as the median. The *M*-estimator concept is introduced by analysing the robustness of two important location estimators, the mean and the median. From a sample of size *n*, both can be obtained by solving two optimisation problems:

9 $$\begin{aligned} \min \sum _{i=1}^{n}(x_{i} - T)^{2} = 0 \end{aligned}$$

for the first estimator and:

10 $$\begin{aligned} \min \sum _{i=1}^{n}|x_{i} - T \vert = 0, \end{aligned}$$

for the second, from which the following equations are obtained:

11 $$\begin{aligned} \sum _{i=1}^{n}(x_{i} - T)&= 0,\nonumber \\ \sum _{i=1}^{n}\textrm{sig}(x_{i} - T)&= 0, \end{aligned}$$

whose solutions are, respectively, the mean and the median of the fitness distribution. Thus, when the distribution is unknown, the sample median is a better estimator of the location parameter than the sample mean.

Robust estimators can be significantly tied to the properties of the function on which they are based. This is discussed below:

- As for the mean, its associated function is the identity, which designates the excessive sensitivity of this estimator to the presence of extreme values in the sample.
- The sample median is based on a bounded nature function, making it a less sensitive estimator to the presence of outliers in the sample.

In terms of obtaining robust estimators of scale, there are multiple proposals. The most classical estimator is, given a random sample of size *n*, $X_{1}, X_{2},\ldots , X_{n}$, to use the median absolute deviation from sample median, *MAD*, defined as $MAD = {|X_{i} - MD |}$; $i = 1, 2,\ldots , n$, where *MD* is the sample median.

Authors in^59^ proposed another standard estimator found in many statistical packages. It is the interquartile range $Q_{3,f} - Q_{1,f}$ (denoted $IRQ_{f}$ for simplicity) where $F(Q_{3,f}) = 3/4$ and $F(Q_{1,f}) = 1/4$, which has a breakdown point of 25%, which is the point after an estimator becomes useless. It is a robustness measurement; the larger the breakdown point, the better the estimator. If an estimator has a high breakdown point, it may be called a resistant statistic.

Discussing breakdowns, *MAD* has the best possible breakdown point of a 50th percentile, where its influence function is bounded, with the sharpest possible limit among all scaling estimators. This property of the *MAD* estimator makes it a better auxiliary scaling estimator than the interquartile range. Nevertheless, it also has disadvantages, as its efficiency on Gaussian distributions is low because it first estimates the *MD* and then assigns equal importance to positive and negative deviations from it. In contrast, the interquartile range does not present this problem because it is not necessary for the quartile to be equally far from the centre. For this reason, the interquartile range has been used in this paper for the M-DSCRO algorithm, detailed in more depth in the next Section.

## M-DSCRO algorithm

This Section describes in more detail the evolutionary stages of the M-DSCRO algorithm, which are similar to those described in “Background and evolutionary stages” section, but it varies in how individuals are selected in the initialisation and reproduction processes.

Both M-SCRO and M-DSCRO algorithms developed in this work avoid the additional parameters configuration in the stages of the evolutionary process. Furthermore, M-DSCRO also checks if the coral reef population follows a normal distribution or not (regarding its fitness), in which case the automatic setting of the parameters varies with respect to M-SCRO algorithm, as can be seen in the following subsection.

### Selection of individuals in M-DSCRO

As aforementioned, M-SCRO assumed that the FL of the population follows a normal distribution. However, it is convenient to think this is not always the case in the evolutionary process. With the M-DSCRO algorithm, during the evolution, whether the FL follows a normal distribution is checked several times. Specifically, a Kolmogorov Smirnov’s test^60^ is used to check the normality of fitness distribution. Based on the theory of robust estimators explained in “Robust estimators” section, the process for selecting corals in the initialisation and reproductive stages of the algorithm varies.

If the distribution is normal, the coral selection process is the same as that established in the M-SCRO algorithm. Otherwise, M-DSCRO uses:

- The median of the fitness at *j*-th generation, $MD_{f_j}$, instead of the mean $\bar{f_j}$ as centralisation estimator. For the sake of simplicity, the notation $C_j$ stands for $\bar{f}_j$ or $MD_{f_j}$, depending on whether the fitness distribution is normal or not, respectively.
- The interquartile range, $IRQ_{f_j}$, instead of the $S_{f_j}$ as scale estimator. For simplicity, notation $SC_j$ stands $S_{f_j}$ or $IRQ_{f_j}$, depending on whether the fitness distribution is normal.

The Kolmogorov Smirnov’s test is calculated only in a certain number of cases:

- When the population is initialised (after generating and evaluating the reef), that is, in the first generation.
- If *G* is the total number of generations, M-DSCRO also applies the Kolmogorov Smirnov’s test in the generations 2*G*/7, 4*G*/7 and 6*G*/7 of the evolutionary process. That is, the statistics selected by the test results in generation 2*G*/7 are used until generation 4*G*/7, and so on. For instance, if the distribution fitness is not normal at generation 2*G*/7, then $C_j = MD_{f_j}$ and $SC_j = IRQ_{f_j}$ is used until generation 4*G*/7 when the test is applied again.
- If the condition for the coral reef to be reset occurs.

The above values are set in this way for two reasons: (1) More checks throughout the evolution have not provided better results, as well as increasing the computational cost, (2) after the initial check, we continue at 2*G*/7 to give the population some time to evolve, do another check about halfway through the evolution, and finish with 6*G*/7 to give the population some time to evolve after doing the third check with the possible change.

### Initialisation in M-DSCRO

M-DSCRO initialises *P* corals on the reef, i.e. *P* random ANNs representing feasible solutions to our problem. In the initialisation phase, instead of using the interval $(\bar{f}_{1} - S_{f_{1}}, 1]$ described in “Initialisation in M-CRO and M-SCRO” section, the interval $(C_1 - SC_1, 1]$ is used ($\rho$ is unnecessary). That is, those corals whose fitness is not in the above interval are eliminated. If $(C_1 - SC_1) \le 0$ no corals are removed from the reef. Algorithm 1 summarises this procedure.

**Algorithm 1:**
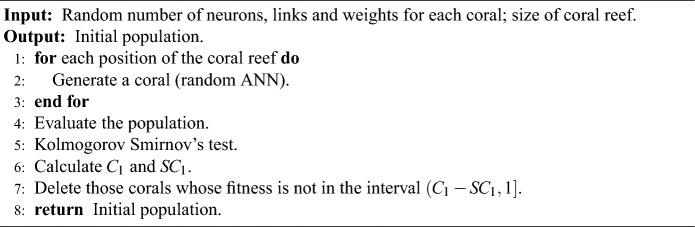
Initialisation algorithm in M-DSCRO.

### Sexual reproduction in M-DSCRO

Following the philosophy of M-CRO and M-SCRO algorithms, there are two types of sexual operators. On the one hand, external sexual reproduction or broadcast spawning must explore the search space. As mentioned in “Crossover operator in artificial neural networks” section, using crossover operators with EANNs has several drawbacks. Therefore, structural mutations are applied for those corals whose fitness function satisfies:

12 $$\begin{aligned} f_{ij} \in (C_j - SC_j, 1]. \end{aligned}$$

On the other hand, the internal sexual reproduction of brooding tries to exploit the search space. In this way, a parametric mutation is applied to the remaining individuals:

13 $$\begin{aligned} f_{ij} \in [0, C_j- SC_j]. \end{aligned}$$

As in the M-CRO and M-SCRO algorithms, each mutated coral becomes part of the candidate solution pool. Also, the *i*Rprop*+* algorithm is applied to the best mutated individual in both broadcast spawning and brooding, and both individuals are added to the pool.

Sexual reproduction procedures are summarised in Algorithm 2.

**Algorithm 2:**
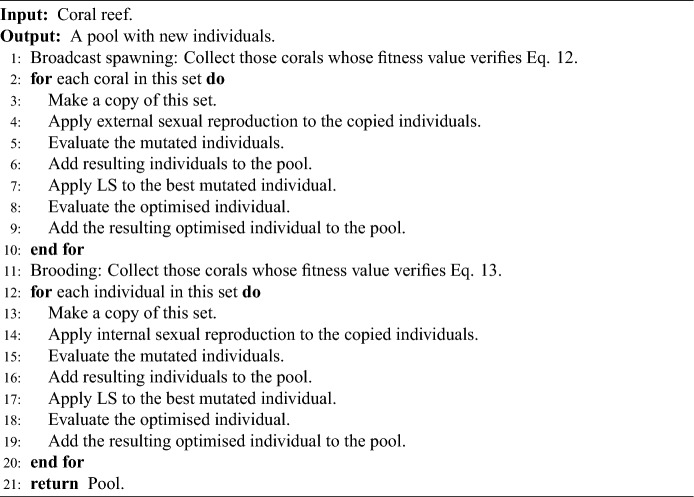
Sexual reproduction process in M-DSCRO.

### Asexual reproduction in M-DSCRO

Asexual reproduction should ensure the survival of one of the best corals. To do this, M-DSCRO selects a set of corals whose fitness function satisfies:

14 $$\begin{aligned} f_{ij} \in (C_j + SC_j, 1]. \end{aligned}$$

If $(C_j + SC_j) \ge 1$, asexual reproduction is not carried out. A random coral has been duplicated from this set at this stage of the reproduction process. In this way, a good coral is kept, but premature algorithm convergence is avoided. Note that $F_a$ parameter is also not needed in M-DSCRO. Algorithm 3 shows this process.

**Algorithm 3:**
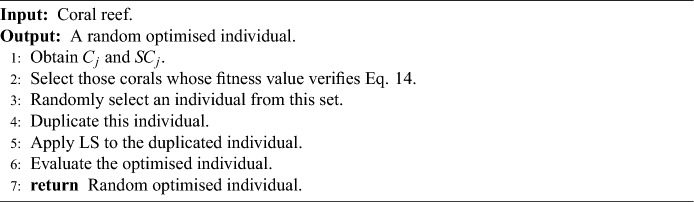
Asexual reproduction process in M-DSCRO.

### Settlement in M-DSCRO

The larvae settlement follows the same structure as in M-CRO and M-SCRO algorithms.

If the settlement is carried out after sexual reproduction, the individuals to be established are in the pool. If the settlement takes place after asexual reproduction, it is the duplicated and optimised individual that tries to establish itself on the reef. Algorithm 4 shows this procedure.

**Algorithm 4:**
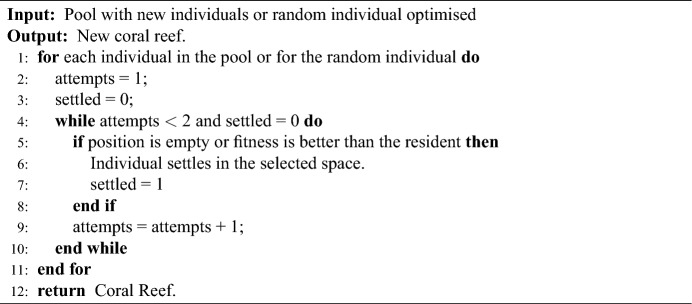
Settlement process in M-DSCRO.

### Depredation in M-DSCRO

The depredation also follows the same structure as in M-CRO and M-SCRO algorithms. It is important to note that in M-DSCRO algorithm, the depredation removes from the reef those individuals whose fitness function is in the interval $[0, C_j - 2SC_j]$. If $(C_j - 2SC_j) \le 0$, depredation is not carried out. Again, the parameters $F_d$ and $P_d$ do not need to be configured. Algorithm 5 shows this procedure.

**Algorithm 5:**
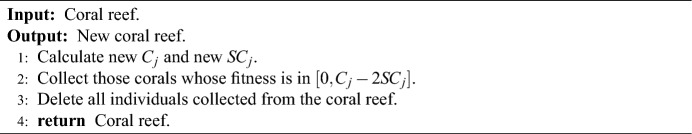
Depredation process in M-DSCRO.

### Reef restarting in M-DSCRO

After each Settlement and Depredation, it is important to check for the possible restart of the coral reef. In the M-DSCRO algorithm, if the restart is applied, it is necessary to verify whether the population is normal. The restart methodology saves the healthiest coral in the reef. The rest of the coral reef is generated randomly as in the Initialisation process. This will be shown in Algorithm 6.

**Algorithm 6:**
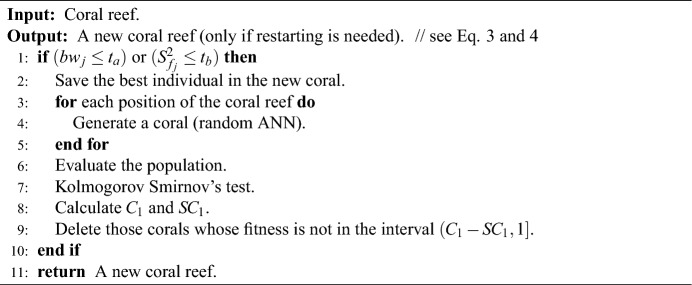
Check reset in M-DSCRO.

### Stop condition in M-DSCRO

Finally, remember that, in the stop condition, in addition to checking if the maximum number of generations has been reached, a Kolmogorov Smirnov’s test is applied for M-DSCRO in the generations 2*G*/7, 4*G*/7 and 6*G*/7 of the evolutionary process for checking if the population is normal or not.

## Experiments

In this section, detailed information will be provided about the datasets used in the experimentation, the ANN model used as individuals, the metrics employed to evaluate the performance of the different algorithms, and the experimental setup of the parameters needed.

### Datasets used in our experiments

In this work, the performance of the M-CRO, M-SCRO and M-DSCRO is evaluated considering a total of 40 datasets for supervised classification problems, whose main characteristics are summarised in Table  1: identifier (*ID*), assigned by ordering the datasets alphabetically, name (*Dataset*), number of patterns (*#Patt.*), characteristics (*#Char.*), classes (*#Classes*), their distribution (*Class Dist.*), the imbalance ratio (*IR*), and the source of information (*Source*). As can be seen, this selection includes various types of classification problems with different fields of application (medical, energy or benchmarks, among others). Also, they cover a wide variety in terms of the number of patterns (from 132 to 19020), number of attributes (2 to 121), classes (2 to 15) and imbalance ratio (1 to 40). The underlying idea is to test the memetic algorithms on a wide variety of datasets and to verify that the dynamic proposal (M-DSCRO), as the paper’s contribution, improves the other two algorithms.

### ANN model

We use MLPs with a hidden layer and Sigmoidal transfer functions (SUs) in the hidden layer, and linear units in the output layer, whose functional model can be represented as:

15 $$\begin{aligned} f_q(\textbf{x})=\beta _{0}^q+\sum _{l=1}^{M}\beta _{l}^qB_{l}(\textbf{x},\textbf{w}_{l}); q=1,2,\ldots ,Q \end{aligned}$$

replacing $$B_{l}(\textbf{x},\textbf{w}_{l})$$ by:

$$\begin{aligned} B_{l}(\textbf{x},\textbf{w}_{l})=\frac{1}{1+exp\left( - \left( w_{0l} + \sum _{k=1}^K w_{kl}x_{k}\right) \right) } \end{aligned}$$

where $\textbf{w}_{l} = (w_{1l}, \ldots , w_{Kl})$ is the vector of weights of the connections between the input layer and the *l*-th hidden node, *M* is the number of sigmoidal units in the hidden layer, *Q* is the number of classes of the problem, *K* is the number of features in each pattern to be classified, $\textbf{x}$ is the input pattern and $B_{l}(\textbf{x},\textbf{w}_{l})$ is the sigmoidal basis function.

Taking the *softmax* activation function into account, represented in Eq. (16), it can be observed that the class predicted by the classifier corresponds to the neuron on the output layer whose a posteriori probability is greater.

16 $$\begin{aligned} g_{q}(\textbf{x})=\frac{\exp f_{q}(\textbf{x})}{\sum _{q=1}^Q\exp f_{q}(\textbf{x})} \text {,} \end{aligned}$$

where $f_{q}(\textbf{x})$ is the output of the *q-th* output neuron for pattern $\textbf{x}$, and $g_{q}(\textbf{x})$ is the probability that pattern $\textbf{x}$ has of belonging to *q-th* class. Therefore, one of the classes does not need to be estimated due to the properties of the probability function.

**Table 1:** Characteristics of the selected classification datasets, sorted alphabetically.

ID	Dataset	#Patt.	#Char	#Classes	Class Dist.	IR	Source
1	Balance Scale	625	4	3	288-49-288	5.88	UCI^61^
2	Breast Cancer Wisconsin (Diagnostic)	569	30	2	357-212	1.68	UCI^61^
3	Breast Cancer Wisconsin (Original)	699	9	2	458-241	1.90	UCI^61^
4	Breast Cancer Wisconsin (Prognostic)	194	32	2	148-46	3.22	UCI^61^
5	Car Evaluation	1728	21	4	1210-384-65-69	18.62	UCI^61^
6	Chess (King-Rook vs. King-Pawn)	3196	38	2	1669-1527	1.09	UCI^61^
7	Connectionist Bench	208	60	2	111-97	1.14	UCI^61^
8	Contraceptive Method Choice	1473	9	3	629-333-511	1.89	UCI^61^
9	Credit-approval	666	46	2	367-299	1.23	UCI^61^
10	Dermatology	366	34	6	112-61-72-49-52-20	5.60	UCI^61^
11	Electrical Grid Stability Simulated Data	10000	12	2	6380-3620	1.76	UCI^61^
12	Eucalyptus	736	91	5	180-107-130-214-105	2.04	Kaggle^62^
13	Fog formation at Valladolid	3340	6	2	2605-735	3.54	Durán et al.^63^
14	Gene Expression	3175	120	3	762-765-1648	2.16	UCI^61^
15	Glass Identification	214	9	6	70-76-17-13-9-29	8.44	UCI^61^
16	Haberman’s Survival	306	3	2	81-225	2.78	UCI^61^
17	Hayes-Roth	132	4	3	51-51-30	1.70	UCI^61^
18	Horse	364	58	3	224-88-52	4.31	UCI^61^
19	Horse-Colic	300	121	2	191-109	1.75	UCI^61^
20	Image-Segmentation	210	19	7	30-30-30-30-30-30-30	1.00	UCI^61^
21	Ionosphere	351	34	2	126-225	1.79	UCI^61^
22	LEV	1000	4	5	93-280-403-197-27	14.93	OpenML^64^
23	Libras Movement	360	90	15	24 patterns in each class	1.00	UCI^61^
24	Liver Disorders	345	6	2	200-145	1.38	UCI^61^
25	Lymphography	148	38	4	2-81-61-4	40.50	UCI^61^
26	MAGIC Gamma Telescope	19020	10	2	12332-6688	1.84	UCI^61^
27	Newthyroid	215	5	3	150-35-30	5.00	UCI^61^
28	Pima Indians Diabetes	768	8	2	500-268	1.87	Kaggle^62^
29	SaltWaterDistortion Dataset	1000	10	4	32-352-399-217	12.47	Senshina et al.^65^
30	Solar Flare	1066	38	6	331-239-211-147-95-43	7.70	UCI^61^
31	South German Credit	1000	61	2	700-300	2.33	UCI^61^
32	Splice-junction Gene Sequences	3190	60	3	767-768-1655	2.16	UCI^61^
33	Statlog (Australian Credit Approval)	690	51	2	307-383	1.25	UCI^61^
34	Statlog (Landsat Satellite)	6435	36	6	1533-703-1358-626-707-1508	2.45	UCI^61^
35	Teaching Assistant Evaluation	151	5	3	49-50-52	1.06	UCI^61^
36	Thoracic Surgery Data	470	27	2	400-70	5.71	UCI^61^
37	Thyroid Disease allbp	2028	23	5	936-716-265-82-29	32.28	UCI^61^
38	Tic-Tac-Toe Endgame	958	27	2	332-626	1.89	UCI^61^
39	Toy	300	2	5	35-87-79-68-31	2.81	Da Costa et al.^66^
40	Waveform Database Generator	5000	40	3	1692-1653-1655	1.02	UCI^61^

Note that some databases have undergone preprocessing, such as removing missing values or binarising categorical variables. This causes these databases’ number of patterns or attributes to vary slightly. However, the final databases will be available in Section Data availability. Within the table, #Patt. represents the number of patterns, #Char. denotes the number of characteristics, #Classes signifies the number of classes present in the dataset, Class Dist. indicates the distribution of classes, and IR refers to the Imbalance Ratio, providing a comprehensive overview of the dataset features. .

### Evaluation metrics

When considering classification problems, the most common metric is the percentage of patterns correctly classified or Correctly Classified Rate (*CCR*), which is defined formally as follows:

17 $$\begin{aligned} CCR=\frac{1}{N} \sum _{q=1}^Q n_{qq}, \end{aligned}$$

where *N* is the number of patterns in the training or generalisation set, and $n_{qq}$ is the number of patterns from the *q*-th class that are correctly classified.

*CCR* is a general approach to assess the goodness of the classification model. However, *CCR* only captures the global accuracy of the model without considering the minority classes in imbalanced datasets. As seen in Table 1, there are many datasets with an imbalanced nature ($IR > 1$), so another metric should be used for comparison purposes. In this sense, the Minimum Sensitivity (*MS*)^45^ is the accuracy rate of the worst classified class. *MS* is defined as follows:

18 $$\begin{aligned} MS=min\left\{ S_{q};q=1,\ldots ,Q\right\} \text {,} \end{aligned}$$

where $S_{q}$ is the percentage of examples correctly predicted as belonging to the *q*-th class. Thus, $S_q = n_{qq} / n_q$, where $n_q$ represents the total count of patterns belonging to this *q*-th class. The use of this metric is based on the fact that it is more directly interpretable than other alternatives considered (such as a multiclass f1-score): by calculating this ratio for the worst classified class (minimum value of the sensitivity), the classifier is ensured to obtain at least the given performance for all classes of the problem.

In this way, we will not only obtain a value of the overall performance of the evaluated algorithms but also how they behave, at least in the class that ranks worst.

However, the error function used during the evolutionary training process of the ANNs is the cross-entropy function, *E*. The *E* error function is a continuous function, which makes the convergence more robust with respect to *CCR*. The values that can take the *E* metric are between 0 and $\infty$:

19 $$\begin{aligned} E = -\left( \frac{1}{N}\right) \sum _{n=1}^{N} \sum _{q=1}^{Q} y_{n}^{q} \log {g_{q} (\textbf{x})}, \end{aligned}$$

where $y_{n}^{q}$ is equal to 1 if the pattern *n* belongs to *q-th* class, and 0 otherwise, and where $g_{q} (\textbf{x})$ is the predicted probability (Eq. 16) that the pattern *n* belongs to class *q*. Finally, the metric is transformed to be maximised in the interval [0, 1] by the expression $A=1/(1+E)$ so that the statistically-driven evolutionary procedure makes sense.

### Experimental setting

Each dataset is divided using a stratified hold-out with 75% of the patterns for training and the remaining 25% for testing. Given the stochasticity of the algorithms, to obtain statistically representative average results, they are run 30 times with different seeds.

A multilayer neural network with a single hidden layer has been shown to be a universal approximator. Thus a shallow network, with a single layer of hidden units has been used, given that it is sufficient to represent any function with the necessary degree of accuracy^67^. For the hidden and output layers, the initialisation of the weights is random, and a bias value needs to be trained for each SU and linear unit. The number of outputs corresponds to the number of classes minus one for a concrete dataset (given that the *softmax* function is used in the output layer).

For the structural mutations, the probability of choosing a type of mutation is equal to 1/5. One or two neurons are added or deleted during these mutations. For adding or deleting links, we randomly add or delete 30% of the links in the input-hidden layers and 5% in the hidden-output layers. Weights are assigned using uniform distribution defined throughout two intervals, [-5,5] for connections between the input layer and hidden layer and [-10,10] for connections between the hidden layer and the output layer. For the parametric mutation, $\alpha _{1}(0) = 0.5$ and $\alpha _{2}(0)=1$. All these parameters values have been taken from previous references^49,50^, which present an EA with similar mutators. Note that, in any case, the use of an EA, which dynamically adapts to the problem evaluated, results in a performance which is negligibly affected by minor changes in these parameters.

Based on the literature^20^, the coral reef size (*P*) has been set to 100 individuals, and the number of settlement attempts is 2 for each individual of the pool. As mentioned above, M-CRO requires a more extensive configuration than its statistical versions. The ratio of free positions on the reef ($\rho$) is set to 0.1, the percentage for the asexual reproduction ($F_a$) is established to 0.05, the percentage for broadcast spawning has been set to 0.75, while the remaining 0.25 are selected for brooding; and finally, the percentage and probability of depredation are 0.05 and 0.1, respectively.

Although the thresholds are configurable for coral reef reset conditions, a threshold value of 2% (0.02) for the $bw_j$ parameter is somewhat acceptable. In the same way, a threshold value of 0.05 for $S_{f_j}^2$ is robust.

For the *iRprop*^+^ algorithm, the number of epochs established is 25 (a more significant number of epochs does not improve the results), $\eta ^+=1.2$, $\eta ^-=0.5$, $\Delta _0=0.0125$ (the initial value of the $\Delta _{ij}$), $\Delta _{\min }=0$ and $\Delta _{\max }=50$ (see^27^ for *iRprop*^+^ parameter descriptions).

For the sake of conciseness, we have chosen to present the results of the memetic versions since they are all better than their standard version, and the same analysis can be extracted.

To further validate the effectiveness of the proposed M-DSCRO method, it was compared against four well-known state-of-the-art methods: C4.5 Decision Tree, Logistic Regression (LR), Multilayer Perceptron (MLP), and Support Vector Machine (SVM). The aim was to surpass these established algorithms in performance. The selection of hyperparameters for each algorithm involved a nested 10-fold cross-validation process, repeated three times on the training dataset, focusing on minimising the cross-validation error. The optimal hyperparameter set, resulting in the lowest cross-validation error, was then applied to the entire training dataset to evaluate the final performance in the test set. The hyperparameter tuning was conducted as follows. For LR, both l1 and l2 penalty functions were considered, with the cost parameter ($C$) ranging from $10^{-3}$ to $10^{3}$. In the case of SVM with a Gaussian kernel, $C$ and the kernel width ($\sigma$) were varied within the same range of $10^{-3}$ to $10^{3}$. The C4.5 Decision Tree’s configuration included the Gini index and entropy as criteria, with the maximum tree depth set between 3 and 6, and the minimum number of samples required at a leaf node ranging from 2 to 10. For the MLP, a single hidden layer was used, with the number of neurons in this layer chosen from the set {2, 4, 6, 8, 10} (similar with respect to our neural networks). The training iterations were set within {500, 1000, 1500}, the learning rate ($\alpha$) was chosen from {0.1, 0.5, 1}, and the momentum ($\mu$) from {0.3, 0.5, 0.7, 0.9}.

## Results and discussion

The results of *CCR* and *MS* are shown in Table 2, in which mean values and standard deviation ($Mean_{std}$) of the 30 runs for each algorithm have been calculated, as well as the average number of neurons ($\#\overline{Neur.}$) and links ($\#\overline{Links}$) used by the best methodology in *CCR*. Also, each method’s mean ranking ($\bar{r}$) has been included, assigning 1 to the best method and 3 to the worst. The best method for each dataset is in bold, while the second one is in italics, considering the two metrics separately.

**Table 2:** Mean and standard deviation values ($Mean_{std}$) of Correct Classification Rate (*CCR*) and Minimum Sensitivity (*MS*) obtained by all the algorithms in each dataset for the 30 runs.

ID	*CCR*			*MS*			$\#\overline{Neur.}$	$\#\overline{Links}$
	M-CRO	M-SCRO	M-DSCRO	M-CRO	M-SCRO	M-DSCRO		
1	$0.9547_{0.0151}$	$0.9671_{0.0139}$	$\mathbf {0.9676_{0.0133}}$	$0.7362_{0.1263}$	$\mathbf {0.8562_{0.0972}}$	$0.8274_{0.1167}$	$9.0000_{0.0000}$	$59.2000_{2.2034}$
2	$0.9462_{0.0189}$	$0.9502_{0.0197}$	$\mathbf {0.9519_{0.0163}}$	$0.9313_{0.0256}$	$\mathbf {0.9398_{0.0246}}$	$0.9362_{0.0266}$	$4.9666_{0.1825}$	$85.7000_{9.6780}$
3	$0.9476_{0.0113}$	$0.9451_{0.0112}$	$\mathbf {0.9491_{0.0116}}$	$0.8866_{0.0319}$	$0.8891_{0.0300}$	$\mathbf {0.8978_{0.0279}}$	$5.0000_{0.0000}$	$65.5333_{5.7459}$
4	$\mathbf {0.7000_{0.0583}}$	$0.6605_{0.0622}$	$0.6735_{0.0514}$	$0.3111_{0.1256}$	$0.3321_{0.1582}$	$\mathbf {0.3540_{0.1687}}$	$5.0000_{0.0000}$	$92.9333_{9.4974}$
5	$0.9745_{0.0129}$	$0.9807_{0.0144}$	$\mathbf {0.9827_{0.0100}}$	$0.8734_{0.0784}$	$0.9126_{0.0690}$	$\mathbf {0.9127_{0.0662}}$	$6.9666_{0.1825}$	$132.2000_{9.8415}$
6	$0.9766_{0.0081}$	$0.9852_{0.0049}$	$\mathbf {0.9854_{0.0067}}$	$0.9662_{0.0097}$	$\mathbf {0.9748_{0.0083}}$	$\mathbf {0.9748_{0.0089}}$	$5.9666_{0.1825}$	$147.1000_{12.4078}$
7	$0.7519_{0.0488}$	$0.7615_{0.0473}$	$\mathbf {0.7623_{0.0476}}$	$0.6236_{0.0830}$	$\mathbf {0.6528_{0.0712}}$	$0.6472_{0.0732}$	$5.6666_{0.6064}$	$163.4333_{28.3994}$
8	$0.5470_{0.0144}$	$0.5405_{0.0114}$	$\mathbf {0.5479_{0.0144}}$	$0.3735_{0.0534}$	$0.3550_{0.0518}$	$\mathbf {0.3739_{0.0837}}$	$5.0000_{0.0000}$	$59.9000_{1.5833}$
9	$0.8469_{0.0157}$	$0.8551_{0.0190}$	$\mathbf {0.8553_{0.0192}}$	$0.8230_{0.0247}$	$0.8317_{0.0314}$	$\mathbf {0.8320_{0.0226}}$	$5.9666_{0.1825}$	$163.8000_{19.7996}$
10	$0.9414_{0.0307}$	$0.9392_{0.0337}$	$\mathbf {0.9516_{0.0237}}$	$0.7362_{0.1235}$	$0.7365_{0.1537}$	$\mathbf {0.7599_{0.1104}}$	$6.5333_{0.6814}$	$149.7333_{19.5958}$
11	$0.9168_{0.0186}$	$\mathbf {0.9406_{0.0045}}$	$0.9400_{0.0030}$	$0.8816_{0.0334}$	$\mathbf {0.9224_{0.0080}}$	$0.9211_{0.0068}$	$5.0000_{0.0000}$	$62.7666_{3.7936}$
12	$0.5667_{0.0326}$	$0.5906_{0.0290}$	$\mathbf {0.5909_{0.0303}}$	$0.2810_{0.1244}$	$0.3502_{0.0775}$	$\mathbf {0.3566_{0.0839}}$	$10.0000_{0.0000}$	$522.0333_{46.9647}$
13	$0.8665_{0.0035}$	$0.8664_{0.0041}$	$\mathbf {0.8668_{0.0045}}$	$0.6780_{0.0121}$	$0.6787_{0.0110}$	$\mathbf {0.6826_{0.0132}}$	$2.8333_{0.4611}$	$17.9000_{3.4775}$
14	$0.8508_{0.0222}$	$\mathbf {0.8727_{0.0132}}$	$\mathbf {0.8727_{0.0130}}$	$0.7721_{0.0394}$	$0.7805_{0.0386}$	$\mathbf {0.7834_{0.0374}}$	$6.9666_{0.1825}$	$504.2666_{34.6658}$
15	$0.7296_{0.0362}$	$0.7415_{0.0470}$	$\mathbf {0.7528_{0.0319}}$	$0.3278_{0.1920}$	$0.3815_{0.1745}$	$\mathbf {0.4173_{0.1733}}$	$9.9666_{0.1825}$	$110.6000_{5.1030}$
16	$0.6917_{0.0269}$	$\mathbf {0.7053_{0.0157}}$	$0.7031_{0.0251}$	$0.2517_{0.0500}$	$0.2517_{0.0482}$	$\mathbf {0.2600_{0.0481}}$	$5.0000_{0.0000}$	$24.6000_{1.0699}$
17	$0.7412_{0.0548}$	$0.7539_{0.0503}$	$\mathbf {0.7667_{0.0551}}$	$0.6029_{0.0673}$	$0.6208_{0.0569}$	$\mathbf {0.6250_{0.0790}}$	$5.0000_{0.0000}$	$33.8333_{2.3056}$
18	$0.6176_{0.0354}$	$0.6425_{0.0414}$	$\mathbf {0.6440_{0.0352}}$	$0.2485_{0.1316}$	$0.2383_{0.1283}$	$\mathbf {0.2915_{0.1214}}$	$5.0000_{0.0000}$	$216.1666_{17.0255}$
19	$0.7084_{0.0385}$	$0.7258_{0.0434}$	$\mathbf {0.7422_{0.0381}}$	$0.5420_{0.0737}$	$0.5346_{0.0800}$	$\mathbf {0.5481_{0.0648}}$	$6.9666_{0.1825}$	$361.8666_{42.4903}$
20	$0.8577_{0.0309}$	$0.8613_{0.0380}$	$\mathbf {0.8685_{0.0379}}$	$0.5500_{0.0963}$	$0.5417_{0.0889}$	$\mathbf {0.5583_{0.1075}}$	$9.9666_{0.1825}$	$156.1000_{13.8945}$
21	$0.9140_{0.0231}$	$0.9170_{0.0252}$	$\mathbf {0.9190_{0.0300}}$	$\mathbf {0.8198_{0.0596}}$	$0.8167_{0.0579}$	$0.7990_{0.0675}$	$5.2000_{0.8469}$	$96.0333_{14.0577}$
22	$0.6051_{0.0258}$	$0.6051_{0.0200}$	$\mathbf {0.6093_{0.0176}}$	$\mathbf {0.0381_{0.0643}}$	$0.0238_{0.0541}$	$0.0333_{0.0720}$	$8.0000_{0.0000}$	$73.2333_{1.7749}$
23	$0.6930_{0.0374}$	$0.6996_{0.0403}$	$\mathbf {0.7115_{0.0473}}$	$\mathbf {0.1833_{0.1103}}$	$0.1500_{0.1265}$	$0.1611_{0.1348}$	$15.0000_{0.0000}$	$744.5666_{97.7702}$
24	$0.7143_{0.0383}$	$0.7221_{0.0354}$	$\mathbf {0.7256_{0.0335}}$	$0.6583_{0.0606}$	$0.6603_{0.0488}$	$\mathbf {0.6745_{0.0455}}$	$5.0000_{0.0000}$	$35.1000_{2.3975}$
25	$0.8000_{0.0548}$	$0.7946_{0.0529}$	$\mathbf {0.8090_{0.0581}}$	$\mathbf {0.1056_{0.2749}}$	$0.0700_{0.2136}$	$0.0250_{0.1369}$	$5.5666_{0.5683}$	$135.0000_{20.6614}$
26	$0.8601_{0.0046}$	$0.8574_{0.0037}$	$\mathbf {0.8604_{0.0028}}$	$0.7374_{0.0089}$	$0.7381_{0.0055}$	$\mathbf {0.7434_{0.0050}}$	$5.0000_{0.0000}$	$49.4666_{4.1996}$
27	$0.9370_{0.0179}$	$0.9444_{0.0154}$	$\mathbf {0.9494_{0.0168}}$	$0.6841_{0.0690}$	$0.6989_{0.0697}$	$\mathbf {0.7254_{0.0931}}$	$7.3666_{0.7648}$	$41.0333_{4.2221}$
28	$0.7698_{0.0229}$	$0.7630_{0.0210}$	$\mathbf {0.7702_{0.0212}}$	$0.5786_{0.0363}$	$0.5647_{0.0487}$	$\mathbf {0.5791_{0.0468}}$	$4.0000_{0.0000}$	$37.9666_{2.1890}$
29	$0.5680_{0.0228}$	$0.5731_{0.0210}$	$\mathbf {0.5739_{0.0170}}$	$\mathbf {0.1542_{0.1563}}$	$0.0855_{0.1144}$	$0.1167_{0.1428}$	$6.0000_{0.0000}$	$82.9333_{2.0499}$
30	$0.7382_{0.0196}$	$0.7417_{0.0137}$	$\mathbf {0.7425_{0.0133}}$	$\mathbf {0.0909_{0.0572}}$	$0.0904_{0.0829}$	$0.0801_{0.0474}$	$8.0000_{0.0000}$	$239.4000_{20.0354}$
31	$0.7076_{0.0232}$	$0.7111_{0.0227}$	$\mathbf {0.7143_{0.0227}}$	$0.4705_{0.1244}$	$\mathbf {0.5031_{0.0838}}$	$0.4738_{0.0776}$	$4.9666_{0.1825}$	$190.1000_{17.0866}$
32	$0.9079_{0.0143}$	$0.9294_{0.0093}$	$\mathbf {0.9296_{0.0137}}$	$0.8613_{0.0278}$	$\mathbf {0.9020_{0.0167}}$	$0.8971_{0.0257}$	$6.0000_{0.0000}$	$953.9666_{84.6911}$
33	$0.8534_{0.0177}$	$0.8543_{0.0244}$	$\mathbf {0.8574_{0.0255}}$	$0.8277_{0.0286}$	$0.8191_{0.0441}$	$\mathbf {0.8306_{0.0430}}$	$4.9000_{0.3051}$	$137.9000_{21.1486}$
34	$0.8787_{0.0053}$	$0.8798_{0.0046}$	$\mathbf {0.8822_{0.0044}}$	$0.5225_{0.0446}$	$0.5316_{0.0265}$	$\mathbf {0.5391_{0.0245}}$	$7.0000_{0.0000}$	$240.8666_{13.3564}$
35	$0.4518_{0.0634}$	$0.4693_{0.0570}$	$\mathbf {0.4702_{0.0577}}$	$0.3342_{0.1006}$	$0.3265_{0.0987}$	$\mathbf {0.3455_{0.1028}}$	$4.0000_{0.0000}$	$32.2666_{1.2298}$
36	$\mathbf {0.8082_{0.0293}}$	$0.8034_{0.0247}$	$0.8003_{0.0252}$	$0.1000_{0.0804}$	$0.1407_{0.0796}$	$\mathbf {0.1593_{0.0997}}$	$4.0000_{0.0000}$	$84.5666_{9.5581}$
37	$0.6249_{0.0113}$	$0.6296_{0.0102}$	$\mathbf {0.6327_{0.0098}}$	$\mathbf {0.0429_{0.0666}}$	$0.0143_{0.0436}$	$0.0143_{0.0436}$	$7.0000_{0.0000}$	$146.1666_{9.1692}$
38	$0.8996_{0.0680}$	$0.9483_{0.0158}$	$\mathbf {0.9535_{0.0167}}$	$0.8402_{0.0885}$	$0.9016_{0.0323}$	$\mathbf {0.9072_{0.0276}}$	$4.0000_{0.0000}$	$77.7000_{6.4921}$
39	$0.8973_{0.0331}$	$0.9040_{0.0304}$	$\mathbf {0.9111_{0.0260}}$	$0.8251_{0.0517}$	$\mathbf {0.8344_{0.0503}}$	$0.8316_{0.0520}$	$8.0000_{0.0000}$	$51.6333_{2.5118}$
40	$0.8565_{0.0074}$	$0.8571_{0.0069}$	$\mathbf {0.8589_{0.0057}}$	$0.8223_{0.0185}$	$0.8185_{0.0138}$	$\mathbf {0.8225_{0.0144}}$	$5.0000_{0.0000}$	$190.0000_{8.4527}$
Mean Rank. ($\bar{r}$)	2.71	*2.15*	**1.14**	2.44	*2.11*	**1.45**		

Average number of neurons ($\#\overline{Neur.}$) and links ($\#\overline{Links}$) used by the best methodology in *CCR*. The mean rankings of all algorithms are also included. The best method for each dataset is in bold, while the second one is in italics.

The proposed method, M-DSCRO, achieves the best results in *CCR*. It obtains the highest value in 36 out of 40 datasets and the second-best value in 3 out of 40 datasets. This demonstrates that the algorithm is capable of achieving excellent global accuracy in almost all databases, showcasing its robustness across various applications and databases with diverse characteristics. The second-best performing algorithm is M-SCRO. It obtains the highest and second-best results in 3 and 30 datasets, respectively. In contrast, M-CRO can only achieve the best value in 2 datasets, indicating its weak performance when compared to the other two methods. These results are consistent with the literature, which shows that SCRO performs better than CRO. Moreover, the dynamic approach enhances their overall performance. The average rankings confirm this analysis, with M-DSCRO having the lowest value (closest to one), and M-SCRO coming in the second position.

Observing the other metric, the algorithms get worse improvement concerning *MS* than *CCR*. This is rather normal as the algorithm is set up to improve entropy, a metric directly related to the percentage of total patterns correctly classified. Nevertheless, the results obtained are undoubtedly reasonable in almost all datasets.

In this sense, it can be observed that the behaviour is similar to that of *CCR* but with more variability. Specifically, the M-DSCRO algorithm has the highest value in 26 out of 40 datasets and is the second-best in 11. Additionally, the differences between M-CRO and M-SCRO are even more minor when looking at this metric. In this case, M-SCRO has the highest *MS* in 8 datasets, while M-CRO is the best one in 7. These results align with the findings of a previous study^41^, where SCRO outperformed CRO, but the difference was not significant. It is worth noting that the mean ranking of M-CRO and M-SCRO (2.44 and 2.11) is much closer when analysing *MS* than when analysing *CCR*. Hence, the statistical version of the algorithm can improve the global accuracy while reducing the number of parameters that need to be determined. However, the accuracy of the worst classified class only improves slightly. Fortunately, the dynamic proposal (M-DSCRO) overcomes this disadvantage by providing a significant improvement, as observed in this analysis.

When exploring stochastic algorithms, the standard deviation of the different runs is an important feature to consider. If the deviations are close to 0, the algorithms are robust and not dependent on random initialisation. This is the case for all three algorithms being evaluated. In addition, the proposed algorithm (M-DSCRO) not only improves in average terms but also reduces the standard deviation in almost all the databases, demonstrating the excellent stability of the algorithm.

In order to analyse the results from a statistical point of view, a set of statistical tests has been used. Firstly, *CCR* values are analysed. A^68^ test has been applied to the *CCR* rankings, which states that, for a level of significance $\alpha = 5\%$, the confidence interval is $C_0 = (0, F_{0.05} = 3.11)$, and the F-distribution statistical value is $F^* = 68.45\notin C_0$. Therefore, the test rejects the null hypothesis stating that all algorithms perform equally in mean ranking of *CCR*. That is, the algorithm effect is statistically significant. Because of this, the best performing method in *CCR* is considered as the control method for a post-hoc test^69^, comparing this algorithm with the other methods. It has been found that comparing all algorithms with a given algorithm (control method) is more sensitive than comparing all algorithms with each other.

The Holm’s test compares the *i*-th and *j*-th algorithms with the following statistic:

$$\begin{aligned} z = \frac{\bar{r}_i - \bar{r}_j}{\sqrt{\frac{k(k+1)}{6N}}}, \end{aligned}$$

where $\bar{r}_i$ is the mean ranking of the *i*-algorithm, *k* is the number of algorithms, and *N* is the number of datasets. With the value of *z*, it is found the probability of a normal distribution and compared it with a level of significance $\alpha$. Holm’s test adjusts the value for $\alpha$ to compensate multiple comparisons, using a procedure that sequentially tests the hypotheses ordered by their significance. The ordered *p*-values are denoted by $p_1, p_2, \dots , p_k$, so that $p_1< p_2<... < p_k$. The test compares each $p_i$ with $\alpha ^*_{i} = \alpha / (k-i)$, starting with the most significant *p*-value. If $p_1$ is lower than $\alpha / (k -1)$, the corresponding hypothesis is rejected, and then it is compared $p_2$ with $\alpha / (k - 2)$, and so on. When a certain null hypothesis is accepted the remaining ones are also accepted.

The results of Holm’s test are reported in Table  3. When using M-DSCRO as the control algorithm (CA), Holm’s test shows that $p_i < \alpha ^*_i$ in all cases, for $\alpha =0.05$, confirming that there are statistically significant differences favouring M-DSCRO. In addition, M-SCRO is statistically better than CRO using *CCR* as a comparison metric (although the differences are lower).

**Table 3:** Holm test results considering M-DSCRO as control algorithm.

i	CA:M-DSCRO		*CCR*	*MS*
	$\alpha ^*_{0.050}$	Algorithm	$p_i$	$p_i$
1	0.025	M-CRO	0.000 (*)	0.000 (*)
2	0.050	M-SCRO	0.000 (*)	0.003 (*)

Its average *CCR* and *MS* is compared to those of M-CRO and M-SCRO: corrected $\alpha$ values, compared methods and *p*-values, all of them ordered by the number of comparison (i). If M-DSCRO results statistically better, it is marked with (*).

Similarly, to determine the existence of statistical differences when comparing *MS*, a Friedman test has been carried out showing that, for a level of significance $\alpha = 5\%$, the F-distribution value obtained is $F^* = 13.23$ which is outside the confidence interval $C_0 = (0, F_{0.05} = 3.11)$. So, again, there are significant differences between the algorithms and, consequently, a Holm’s test has been run with M-DSCRO as CA. The results presented in Table 3 confirm that M-DSCRO is statistically better than the other two methods. Furthermore, in this case, there are no significant differences between M-CRO and M-SCRO, as previously suggested.

### Examining the imbalance ratio

**Figure 3:**
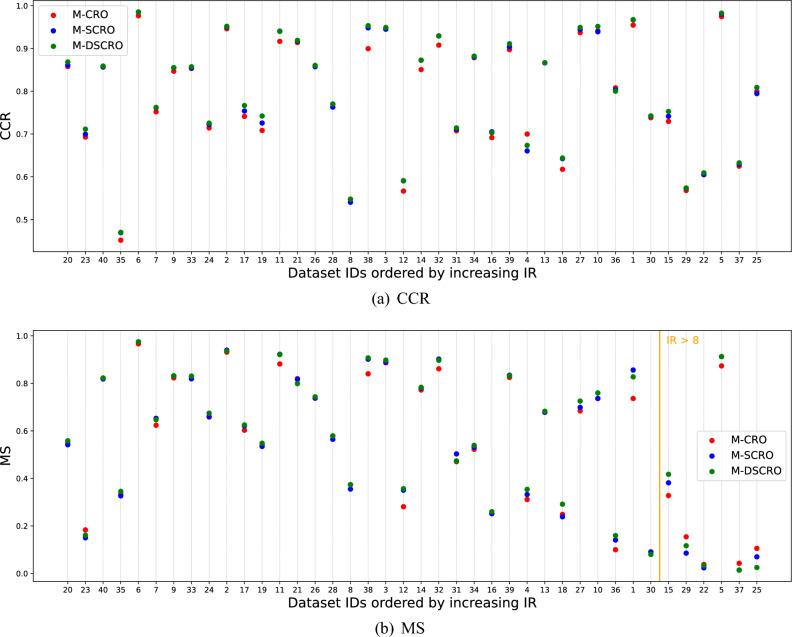
Performance in *CCR* (**a**) and *MS* (**b**) of the three algorithms on the databases sorted in increasing order of IR: M-CRO (red), M-SCRO (blue) and M-DSCRO (green).

A range of imbalanced datasets were worked with as part of the experimental validation. As stated, for each classification dataset, the *IR* has been calculated as the ratio of the number of patterns in the majority class to the number of patterns in the minority class. This information is reported in the column *IR* in Table 1. Furthermore, Fig. 3 shows a graph summarising the performance in *CCR* (a) and *MS* (b) with the databases sorted in increasing order of IR, which facilitates the discussion of the results by analysing this characteristic.

After sorting the databases based on their *IR* and analysing the results, we noticed that for the *CCR*, the M-DSCRO algorithm outperforms the rest in almost all databases, regardless of imbalance.

When studying the *MS*, it is observed that the algorithm M-DSCRO performs the best in classifying the minority classes of both balanced and imbalanced databases. However, for extremely imbalanced databases with *IR* greater than eight, those located to the right of the orange vertical line in Fig. 3b, M-DSCRO is the best in two out of six, with M-CRO performing better in the remaining four. This suggests that while M-SCRO improves global performance for extremely imbalanced databases, it worsens the performance in minority classes. However, this issue is partly resolved with the proposed dynamic version of the algorithm.

### Examining the number of classes

**Figure 4:**
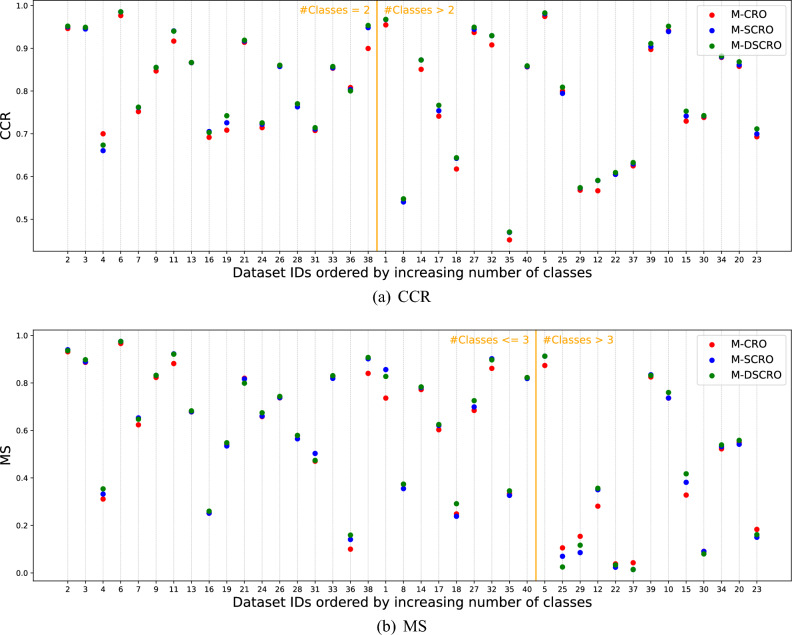
Performance in *CCR* (**a**) and *MS* (**b**) of the three algorithms on the databases sorted in increasing order of number of classes: M-CRO (red), M-SCRO (blue) and M-DSCRO (green).

Also, the datasets cover a wide range in terms of the number of classes, which range from 2 to 15, and are listed in column (*#Classes*) in Table 1. As in the previous part, Fig. 4 shows the CCR (a) and MS (b) performance of the three algorithms on the datasets sorted in ascending order by the number of classes.

Regardless of the number of classes, M-DSCRO performs equally well in *CCR*. Nonetheless, it can be observed that the few times it could be better is in databases with 2 classes (those to the left of the orange line in Fig. 4a). This implies that the dynamic methodological approach excels in more complex databases in terms of overall accuracy.

In *MS*, the M-DSCRO algorithm performs very well for 2 and 3 classes (see vertical orange line in the Fig. 4b). However, when it comes to a larger number of classes, the algorithm is equally competent as M-CRO, while M-SCRO performs the worst. In other words, M-SCRO reduces the performance as the number of classes increases. Therefore, thanks to the dynamic approach, the statistical version becomes more potent without compromising performance for databases with few classes, solving its disadvantages.

### Examining the size

A final analysis has been conducted to check whether the database size affects the results obtained. For this purpose, the number of patterns, attributes, and total size considered as the product of both have been studied.

However, a relationship has yet to be found for any of these elements since the best results of the algorithms are not concentrated in small or large databases but are distributed regardless of size.

### Comparison with state-of-the-art models

In this phase, the performance of the newly introduced M-DSCRO technique is evaluated against other state-of-the-art machine learning models, including C4.5 decision trees, Logistic Regression (LR), Multilayer Perceptron (MLP), and Support Vector Machines (SVM). Table 4 summarises the mean (*Mean*) values of *CCR* and *MS* (we omit the standard deviation to improve the readability of the results). As previously, an average ranking $\bar{r}$ for each approach is provided, where 1 represents the top-performing method, and 5 is the least effective. The leading method for every dataset is highlighted in bold, and the runner-up is denoted in italics, with both metrics evaluated independently.

Regarding *CCR* metric, the M-DSCRO algorithm proposed herein secures the highest performance across 18 databases and attains the second-highest performance in 9 others, as evidenced by its mean rank ($\bar{r}$) of 2.15, positioning it as the foremost method. Following closely is the SVM model, which leads in 11 databases, culminating in an average rank of 2.7. This performance underscores the proposal competitive edge in overall accuracy when juxtaposed with state-of-the-art methods.

Furthermore, according to the *MS* metric, the M-DSCRO algorithm outperforms others, securing the top position in 18 datasets and the second-highest in 9, with an impressive average rank of 2.03. In this scenario, a tie for second place emerges between the C4.5 and LR models, each with an average ranking of 2.96, demoting the SVM model to the fourth position. This indicates that our method maintains superior performance across the board, including for minority classes, whereas the SVM model, despite its overall effectiveness, falls short in adequately addressing minority classes, which are often of paramount importance.

**Table 4:** Mean values of Correct Classification Rate (*CCR*) and Minimum Sensitivity (*MS*) obtained by all the algorithms in each dataset.

ID	*CCR*					*MS*				
	C4.5	LR	MLP	SVM	M-DSCRO	C4.5	LR	MLP	SVM	M-DSCRO
1	0.7201	*0.8590*	0.8568	0.8419	**0.9676**	0.0000	0.0000	0.0000	0.0000	**0.8274**
2	0.9291	*0.9362*	0.6527	0.8865	**0.9519**	0.8679	*0.9057*	0.1107	0.8491	**0.9362**
3	0.9540	**0.9655**	*0.9598*	*0.9598*	0.9491	**0.9474**	*0.9333*	0.9139	*0.9333*	0.8978
4	**0.7500**	**0.7500**	**0.7500**	**0.7500**	0.6735	0.0000	0.0000	0.0000	0.0000	**0.3540**
5	0.9536	0.9281	0.9435	**0.9977**	*0.9827*	0.7647	0.8542	0.5988	**0.9896**	*0.9127*
6	*0.9862*	0.9624	0.9474	**0.9950**	0.9854	*0.9816*	0.9423	0.9322	**0.9921**	0.9748
7	0.7460	0.4921	0.5409	**0.7857**	*0.7623*	0.4348	0.3043	0.2159	*0.6364*	**0.6472**
8	*0.5504*	0.5150	0.4815	**0.5858**	0.5479	**0.4458**	0.3012	0.0643	0.2771	*0.3739*
9	**0.8554**	**0.8554**	0.6458	0.7048	0.8553	0.7935	*0.8261*	0.4862	0.7027	**0.8320**
10	0.4891	**0.9599**	0.7926	0.8942	*0.9516*	0.0000	**0.8478**	0.2758	*0.8108*	0.7599
11	0.8165	0.8016	0.8798	**0.9524**	*0.9400*	0.7362	0.7981	0.8167	**0.9322**	*0.9211*
12	0.5502	0.5633	0.2250	*0.5805*	**0.5909**	0.3120	0.3210	0.0000	*0.3450*	**0.3566**
13	0.8513	**0.8679**	0.7348	0.8081	*0.8668*	0.5387	*0.6625*	0.0156	0.4149	**0.6826**
14	*0.8928*	**0.8979**	0.8852	0.5914	0.8727	0.8063	**0.8691**	*0.8168*	0.0052	0.7834
15	0.6731	0.6346	0.4987	*0.7115*	**0.7528**	**0.5000**	0.2500	0.0000	0.2500	*0.4173*
16	**0.7467**	0.7336	*0.7394*	0.7336	0.7031	**0.3279**	0.0000	0.0612	0.0000	*0.2600*
17	*0.6804*	0.3814	0.4210	0.4845	**0.7667**	*0.1842*	0.0000	0.0976	0.0000	**0.6250**
18	0.5956	0.6140	*0.6229*	0.6140	**0.6440**	0.0000	0.0000	*0.0221*	0.0000	**0.2915**
19	**0.8304**	0.7813	0.7662	*0.7857*	0.7422	**0.6951**	*0.6585*	0.6073	0.5488	0.5481
20	0.2876	*0.8301*	0.1723	0.4641	**0.8685**	0.0000	**0.5909**	0.0000	0.0000	*0.5583*
21	0.9080	0.8736	0.9015	**0.9655**	*0.9190*	*0.8710*	0.6774	0.7333	**0.9032**	0.7990
22	0.5234	0.5113	0.4932	*0.5688*	**0.6093**	0.0000	**0.3000**	0.0000	*0.2500*	0.0333
23	0.1152	0.0632	0.0996	*0.4015*	**0.7115**	0.0000	0.0000	0.0000	0.0000	**0.1611**
24	0.5581	0.6473	0.5536	*0.6550*	**0.7256**	0.4815	*0.6200*	0.3272	0.4907	**0.6745**
25	0.7589	*0.7878*	0.7800	0.7206	**0.8090**	0.0150	0.0200	*0.0210*	0.0150	**0.0250**
26	*0.8351*	0.7902	0.8175	0.8194	**0.8604**	*0.6597*	0.5584	0.6043	0.6316	**0.7434**
27	0.8750	0.9125	*0.9425*	0.9313	**0.9494**	0.5000	*0.7391*	**0.7637**	0.6957	0.7254
28	0.7068	*0.7749*	0.6698	**0.7958**	0.7702	**0.7040**	0.5152	0.1227	0.5606	*0.5791*
29	0.4646	*0.5434*	0.4206	*0.5434*	**0.5739**	**0.1667**	0.1250	0.0000	**0.1667**	0.1167
30	0.5345	*0.7302*	0.6523	0.5521	**0.7425**	0.0000	**0.2676**	0.0000	0.0000	*0.0801*
31	0.6988	**0.7590**	0.6988	0.6988	*0.7143*	0.0000	*0.4667*	0.0000	0.0000	**0.4738**
32	**0.9511**	0.8143	*0.9395*	0.6386	0.9296	**0.9469**	0.5759	*0.9036*	0.2448	0.8971
33	0.8253	*0.8355*	*0.8355*	0.8102	**0.8574**	0.8100	*0.8210*	*0.8210*	0.8000	**0.8306**
34	0.8254	0.7850	0.3914	**0.9142**	*0.8822*	*0.5924*	0.1720	0.0000	**0.6497**	0.5391
35	**0.5405**	0.4324	0.3955	0.4324	*0.4702*	**0.3846**	0.2308	0.1716	0.2727	*0.3455*
36	0.8291	**0.8462**	**0.8462**	**0.8462**	0.8003	*0.0556*	0.0000	0.0000	0.0000	**0.1593**
37	*0.5105*	0.4355	0.4632	0.4618	**0.6327**	0.0000	0.0000	0.0000	0.0000	**0.0143**
38	0.8131	*0.9707*	0.7417	**0.9749**	0.9535	0.6492	**0.9680**	0.5792	**0.9680**	0.9072
39	0.8514	0.3108	0.3036	*0.8919*	**0.9111**	0.7727	0.0000	0.0000	*0.8125*	**0.8316**
40	0.7130	**0.8640**	0.8572	*0.8616*	0.8589	0.6815	**0.8470**	*0.8307*	0.7752	0.8225
Mean Rank. ($\bar{r}$)	3.20	3.06	3.89	*2.70*	**2.15**	*2.96*	*2.96*	3.89	3.16	**2.03**

The mean rankings of all algorithms are also included. The best method for each dataset is in bold, while the second one is in italics.

As with previous analyses, to assess whether significant statistical differences exist in the performance metrics *CCR* and *MS*, two Friedman tests were conducted. These tests revealed that at a significance level of $\alpha = 5\%$, the F-distribution values achieved were $F^* = 7.67$ for CCR and $F^* = 8.37$ for MS. Both values exceed the bounds of the confidence interval $C_0 = (0, F_{0.05} = 2.43)$, indicating significant disparities among the evaluated methods. Consequently, Holm’s post-hoc test was applied to each metric with the M-DSCRO method serving as the control algorithm, and the findings are compiled in Table 5.

**Table 5:** Holm test results considering M-DSCRO as control algorithm.

i	CA:M-DSCRO	*CCR*		*MS*	
	$\alpha ^*_{0.050}$	Algorithm	$p_i$	Algorithm	$p_i$
1	0.013	MLP	0.000 (*)	MLP	0.000 (*)
2	0.017	C4.5	0.003 (*)	SVM	0.001 (*)
3	0.025	LR	0.010 (*)	C4.5	0.008 (*)
4	0.050	SVM	0.119	LR	0.008 (*)

Its average *CCR* and *MS* is compared to those of C4.5, LR, MLP and SVM: corrected $\alpha$ values, compared methods and *p*-values, all of them ordered by the number of comparison (i). If M-DSCRO results statistically better, it is marked with (*).

Although the M-DSCRO algorithm exhibits superior performance in terms of *CCR*, the Holm’s test indicates that its advantage over the SVM model is not statistically significant, whereas significant disparities are observed when compared to C4.5, LR, and MLP. In contrast, the *MS* metric clearly demonstrates the algorithm’s superior performance relative to other methods, with the significant differences being unmistakably pronounced. Based on these findings, the adoption of the M-DSCRO methodology for classification problems is confidently endorsed.

## Conclusions

This paper proposes three memetic algorithms for training and optimising the topology and weights of ANNs simultaneously. Concretely, CRO and its statistical version, SCRO, have been implemented and adapted using suitable operators for this purpose, resulting in M-CRO and M-SCRO algorithms. Also, an improved version of M-SCRO has been proposed in which the hypothesis of normal fitness distribution is tested during the evolution, motivated by the theory of robust estimators. In this way, the algorithm dynamically selects the intervals based on the centralisation and scaling calculated estimators, resulting in the so-called M-DSCRO. The proposed algorithms are finally evaluated on 40 classification datasets by comparing the Correct Classification Rate *CCR*, which measures the accuracy of a classification model by determining the proportion of correctly classified instances, and Minimum Sensitivity *MS*, which is the lowest sensitivity computed across all sensitivities of the problem.

The results show that M-SCRO statistically improves on M-CRO in terms of *CCR* but is equal in terms of *MS*. However, M-SCRO does not require parameter tuning. The new proposed M-DSCRO methodology, however, outperforms the other two algorithms by achieving the same advantage as M-SCRO, i.e. eliminating the need for manual parameter value adjustment based on a dynamic update of the parameters during evolution considering robust estimators and avoiding the assumption of normality of the fitness distribution. According to the study performed, the results of M-DSCRO are significantly better in terms of *CCR* and *MS*. The M-DSCRO algorithm demonstrated superior *CCR* in diverse imbalance levels and performed well in *MS* across balanced and imbalanced datasets. However, in cases of high imbalance (*IR*> 8), M-CRO outperformed M-DSCRO in minority class classification in some instances, a gap partially bridged by its dynamic version. The datasets varied from 2 to 15 classes, with M-DSCRO showing consistent *CCR* performance and excelling in datasets with fewer classes. While effective in *MS* for 2 and 3 classes, its performance aligned with M-CRO as class number increased, with M-SCRO effectiveness waning with more classes. The dynamic approach of M-DSCRO addressed its limitations without affecting its performance in smaller datasets. An analysis on the influence of database size found no clear link between database dimensions and algorithm performance. Finally, a comparison against four state-of-the-art algorithms shows how M-DSCRO excels, proving its superior effectiveness in terms of both overall performance and minority class performance.

For future lines of research, the authors plan to extend this work by using the *CCR* along with *MS* in a multiobjective evolutionary algorithm. It has been shown in^45^ that both objectives are opposite, especially at certain levels. At the beginning of a learning or evolutionary process, *CCR* and *MS* could be cooperative, but after a certain level, objectives become competitive and an improvement in one objective tends to involve a decrease in the other one. *MS* can be considered a complementary measure of *CCR* whose value must be maximised. It will improve *CCR* as a weighted average of the correct classification rates of the *Q* classes. In this way, the pair (*CCR*, *MS*) tries to find a point between the scalar accuracy measure and the multidimensional ones based on misclassification rates.

## Data Availability

The datasets utilised are available at https://www.uco.es/grupos/ayrna/datasets/datasetsMDSCRO.zip. The instructions regarding the format and its usage can be found on the same page https://www.uco.es/grupos/ayrna/index.php/en/investigacion-y-difusion/partitions-and-datasets.
