# Author Correction: A memetic dynamic coral reef optimisation algorithm for simultaneous training, design, and optimisation of artificial neural networks

**DOI:** 10.1038/s41598-024-61717-9

**Published:** 2024-05-14

**Authors:** Francisco Bérchez-Moreno, Antonio M. Durán-Rosal, César Hervás Martínez, Pedro A. Gutiérrez, Juan C. Fernández

**Affiliations:** 1 Department of Computer Science and Numerical Analysis, University of Córdoba, Córdoba, Spain; 2 Department of Quantitative Methods, Universidad Loyola Andalucía, Córdoba, Spain; 3 Maimonides Biomedical Research Institute of Córdoba, IMIBIC, University of Córdoba, 14071 Córdoba, Spain

Correction to: *Scientific Reports* 10.1038/s41598-024-57654-2, published online 23 March 2024

The original version of this Article contained errors in the Acknowledgements section.

“This work has been partially subsidised by “Agencia Española de Investigación (España)” (grant reference: PID2020-115454GB-C22 / AEI / 10.13039 / 501100011033), by “Investigo 2021 Programme”, funded by the European Union, through the “Plan de Recuperación, Transformación y Resiliencia (PRTR)”, and via the Andalusian Department of Employment, Enterprises & Self-Employed - Junta de Andalucía (grant Ref. INVEST_SAE22_004), by Test and Experiment Facilities for the Agri-Food Domain (AgriFoodTEF). Funding body: EU Commission, DIGITAL-2022-CLOUD-AI-02. PI Spanish node: R. García-González (Univ. de Lleida) Dates: 01/01/2023 to 31/12/2027. (grant reference: 101100622). Amount granted to UCO: 742,152€ (+ 2,500,000€ from the MAPA Spanish Ministry) and by ENIA International Chair in Agriculture University of Córdoba. Funding body: MINECO (Cátedras ENIA), Spain. IP: R. Gallardo (UCO). Dates: 01/01/2023 to 31/12/2027. (grant reference: TSI-100921-2023-3) Amount granted: 1,200,000€.”

now reads:

“This work has been partially subsidised by “Agencia Española de Investigación (España)” (grant reference: PID2020-115454GB-C22 / AEI / 10.13039 / 501100011033); by “Investigo 2021 Programme”, funded by the European Union, through the “Plan de Recuperación, Transformación y Resiliencia (PRTR)”, and via the Andalusian Department of Employment, Enterprises & Self-Employed - Junta de Andalucía (grant Ref. INVEST_SAE22_004); by Test and Experiment Facilities for the Agri-Food Domain (AgriFoodTEF), funding body: EU Commission, DIGITAL-2022-CLOUD-AI-02 (grant reference: 101100622); and by ENIA International Chair in Agriculture University of Córdoba, funding body: MINECO (Cátedras ENIA) (grant reference: TSI-100921-2023-3).”

The original Article has been corrected.

